# CD209/CD14^+^ Dendritic Cells Characterization in Rheumatoid and Psoriatic Arthritis Patients: Activation, Synovial Infiltration, and Therapeutic Targeting

**DOI:** 10.3389/fimmu.2021.722349

**Published:** 2022-01-12

**Authors:** Viviana Marzaioli, Mary Canavan, Achilleas Floudas, Keelin Flynn, Ronan Mullan, Douglas J. Veale, Ursula Fearon

**Affiliations:** ^1^ Rheumatology EULAR Centre of Excellence, Centre for Arthritis & Rheumatic Diseases, St Vincent’s University Hospital, University College Dublin, Dublin, Ireland; ^2^ Molecular Rheumatology, School of Medicine, Trinity Biomedical Sciences Institute, Trinity College Dublin, Dublin, Ireland; ^3^ Department of Rheumatology, Tallaght University Hospital, Dublin, Ireland

**Keywords:** monocyte-derived dendritic cells (MoDC), rheumatoid arthritis, psoriatic arthritis, cell development, inflammation, JAK - STAT signaling pathway

## Abstract

Dendritic cells (DC) have a key role in the initiation and progression of inflammatory arthritis (IA). In this study, we identified a DC population that derive from monocytes, characterized as CD209/CD14^+^ DC, expressing classical DC markers (HLADR, CD11c) and the Mo-DC marker (CD209), while also retaining the monocytic marker CD14. This CD209/CD14^+^ DC population is present in the circulation of Healthy Control (HC), with increased frequency in Rheumatoid Arthritis (RA) and Psoriatic arthritic (PsA) patients. We demonstrate, for the first time, that circulatory IA CD209/CD14^+^ DC express more cytokines (IL1β/IL6/IL12/TNFα) and display a unique chemokine receptor expression and co-expression profiles compared to HC. We demonstrated that CD209/CD14^+^ DC are enriched in the inflamed joint where they display a unique inflammatory and maturation phenotype, with increased CD40 and CD80 and co-expression of specific chemokine receptors, displaying unique patterns between PsA and RA. We developed a new protocol of magnetic isolation and expansion for CD209^+^ DC from blood and identified transcriptional differences involved in endocytosis/antigen presentation between RA and PsA CD209^+^ DC. In addition, we observed that culture of healthy CD209^+^ DC with IA synovial fluid (SF), but not Osteoarthritis (OA) SF, was sufficient to induce the development of CD209/CD14^+^ DC, leading to a poly-mature DC phenotype. In addition, differential effects were observed in terms of chemokine receptor and chemokine expression, with healthy CD209^+^ DC displaying increased expression/co-expression of CCR6, CCR7, CXCR3, CXCR4 and CXCR5 when cultured with RA SF, while an increase in the chemokines CCR3, CXCL10 and CXCL11 was observed when cultured with PsA SF. This effect may be mediated in part by the observed differential increase in chemokines expressed in RA *vs* PsA SF. Finally, we observed that the JAK/STAT pathway, but not the NF-κB pathway (driven by TNFα), regulated CD209/CD14^+^ DC function in terms of activation, inflammatory state, and migratory capacity. In conclusion, we identified a novel CD209/CD14^+^ DC population, which is active in the circulation of RA and PsA, an effect potentiated once they enter the joint. Furthermore, we demonstrated that JAK/STAT inhibition can be used as a therapeutic strategy to decrease the inflammatory state of the pathogenic CD209/CD14^+^ DC.

## Introduction

Inflammatory arthritis (IA), including rheumatoid arthritis (RA) and psoriatic arthritis (PsA), are chronic systemic autoimmune diseases that affect the joints. While the two diseases have many common clinical manifestations, there are significant pathogenic differences at a clinical, anatomical, cellular and molecular level, that may explain differential disease outcomes, prognoses and response to treatments (1). While numerous studies have characterized the dysregulated angiogenesis, leukocyte infiltration and proliferation of the lining layer, leading to the destruction of cartilage and bone in both arthropathies (2–10), little is known about the specific contribution of dendritic cells (DC) to the pathogenesis of RA and PsA. Most studies to date have focused on peripheral blood populations due to the rarity and complexity of phenotyping DC from the joint. DCs are a heterogeneous population of professional antigen-presenting cells at the interface of innate and adaptive immunity and are classified according to their tissue location and function. The advance of new technology, including systems immunology and single-cell analysis, has aided scientists to identify new DC subsets in humans, therefore to date, five distinct DC subsets are known to be present in human: the pDC, the classical myeloid subsets (mDC), cDC1 (CD141^+^) and cDC2 (CD1c^+^), the DC3 and the monocyte-derived DC population (11, 12). The CD141^+^ and the CD1c^+^ DC cells are in a semi-mature stage in circulation in IA patients and are enriched at the site of inflammation, where they are further activated (13–16). In animal models, the presentation of collagen-derived peptides by mature DCs has been proven to be sufficient for the induction of RA in mice (17). Wehr and colleagues, have proposed a new working model for the initiation of RA pathogenesis in humans that is driven by DC-T cell interaction, where mature DCs expressing MHC II, prime autoantigen‐specific CD4^+^ T cells, leading to germinal center formation and B cell maturation/autoantibodies production (18). Previous studies by Jongbloed and colleagues demonstrated that mDCs are decreased in the blood of RA patients in comparison to healthy controls (HC) and pDCs were decreased in both RA and PsA, with increased frequency of both subsets observed in IA synovial fluid (13). Consistent with this study, it is now believed that DC are increased in the joint of IA, but not osteoarthritis, patients (19, 20). Previously, we and others have reported the enrichment of myeloid DC, including CD141^+^ DC (14) and CD1c^+^ (15, 21, 22) in the IA joint, which display distinct transcriptional signatures, activation markers and functional capacity to activate T-cells compared to their blood counterparts. Together with the more classical mDCs and the pDCs, a third subset of DCs has been identified, which are derived from monocytes known as ‘Inflammatory Mo-DC’ (14). While many studies have shown the ability of monocytes to differentiate into Mo-DC *in-vitro* (23–25) and *in-vivo* animal studies (26–28), their distinct role and function is still unclear, due to the rarity and lack of definitive markers, however studies to date suggest Mo-DC play a complementary role to conventional DC (CD141 and CD1c) in response to inflammation or infection (11). We have recently shown that RA monocytes *ex-vivo* were phenotypically different to PsA, displaying a more mature phenotype associated with differential rates of differentiation to DC, altered cellular-morphology and early dendrite formation, suggesting that RA monocytes are possibly primed to differentiate into DC (23). However, to date, only a few studies have investigated the DC population derived from monocytes in an *ex-vivo* model. In this study, we identified a novel DC population, deriving from monocytes in the blood of both RA and PsA patients, which we characterize as ‘CD209/CD14^+^ DC’, expressing the classical DC markers, including HLA-DR and CD11c (29, 30), CD209 (DC-SIGN), which are used to identify Mo-DC *in vitro* and *in vivo* (23, 28, 31), while still retaining the monocytic marker CD14 (32). We observed accumulation of these DC in the IA joint, which display a unique inflammatory and maturation phenotype. Importantly, we developed a novel protocol of magnetic isolation and expansion for CD209^+^ DC from blood and observed transcriptional differences between circulatory CD209^+^ DC from RA *vs* PSA patients. Finally, we demonstrated that the JAK/STAT pathway, but not the NF-κB pathway (driven by TNFα), altered CD209/CD14^+^ DC function in terms of activation, inflammatory state and migrative capacity.

## Methods

### Patient Recruitment and Sample Collection

Blood was obtained from patients with active inflammatory arthritis (RA n=62, PsA n=37, OA=6) through the Rheumatology Departments, St. Vincent’s University Hospital or Tallaght Hospital, Dublin, Ireland. Blood was obtained from anonymous healthy donors (HC) as controls from St. Vincent’s University Hospital. Blood samples from both HC and patients were collected in lithium heparin tubes. Synovial fluid and synovial tissues were obtained at arthroscopy or rheumatology clinics (St. Vincent’s University Hospital and Tallaght Hospital, Dublin, Ireland). Ethics for this study was approved by the St. Vincent’s University Hospital and Tallaght University Hospital Ethics and Medical Research Committees and was performed in accordance with the Declaration of Helsinki. All patients gave fully informed written consent. Patient’s demographics are summarized in **Table 1**. 

**Table 1:** Patients’ demographic. Age, Gender, CRP, VAS, serology and detail on treatment. Healthy control (HC) demographic, age and gender.

Parameters	HC (n=ll')	RA (n=&Z)	PsA (fn=37)	OA (n-6)
Age (Mean ± SD)	33±10	55±13.6	51±11.9	67±8
**Gender**				
Female	78%	68%	65%	33%
Male	22%	32%	38%	67%
**CRP**		14+14.1	8+9.3	3.3+3.2
**VAS**		52±30	49±25	50±10
**ACPA**				
POS		60%	0%	0%
NEG		40%	100%	100%
**RF**				
POS		65%	0%	0%
NEG		35%	100%	100%
**Treatments**				
No Medication		27%	35%	100%
Methotrexate alone		10%	19%	0%
MTX in ombination		19%	11%	0%
Anti-TNF		3%	5%	0%
Biological		10%	16%	0%
Others		31%	14%	0%

### PBMC Isolation and Synovial Tissue Digestion

Peripheral blood and synovial fluid -mononuclear cells (PBMC and SFMC, respectively) were isolated by density gradient centrifugation (Lymphoprep, Stemcell Technologies) according to manufacturer’s recommendations. Synovial tissue biopsies obtained at the time of arthroscopy were mechanically and enzymatically digested using the GentleMacs dissociator and a soft tumour dissociation kit (Millenia Biotech, Germany), according to manufacturer’s instructions to yield a single cell suspension of synovial tissue cells. Following arthroscopy, synovial tissue was sectioned into small pieces and added to an enzyme mix composed of 4.7 mL serum-free and antibiotic-free RPMI medium, 200μL of enzyme H, 100μL of enzyme R and 25μL of enzyme A in a gentleMACS C tube. Using the GentleMACS program; m_spleen_4, mechanical stress was applied to the synovial tissue for 60 sec. Samples were then incubated at 37°C for 30 min under constant rotation using the MACSmix Tube Rotator (Miltenyi Biotech). The samples were subsequently exposed to a second mechanical agitation using the m_brain_03 gentleMACS program and incubated for a further 30 min at 37°C. A final mechanical agitation was applied for 30 sec using the gentleMACS h_tumor_03 program. The resulting cell suspension was passed through a 70 μm cell strainer to remove any undigested clumps. Red blood cells were removed using Pharmlyse red blood cell lysis buffer (BD) (33).

### Flow Cytometry

CD209 dendritic cell-derived monocytes (CD209/CD14^+^ DC), were identified by multiparameter flow cytometry analysis. Cells were gated based on forward and side scatter characteristic and doublets were removed. Live Dead Red or Near IR (Molecular Probes) were used to eliminate dead cells. To eliminate non-specific binding of antibodies to Fc-gamma receptors (FcγR), samples were blocked with a human FcγR-binding inhibitor prior to antibody staining (Biolegend). The following antibodies were used in combination to identify the CD209/CD14^+^ DC population and investigate surface markers expressed by cells: CD209 FITC (BD Bioscience), PerCP/Cy5.5 or PE (Biolegend) (Clone DCN46), CD14 PE (Biolegend) or Brilliant Violet 510 (Biolegend) (Clone M5E2), CD80 APC700 (BD Bioscience) (Clone L307.4), CD86 FITC (BD Bioscience) (Clone 2331), CD83 BV711 (clone HB15e), CD40 BV605 (Biolegend) (Clone 5C3), HLA-DR Brilliant Violet 785 or 421 (Biolegend) (Clone G46-6), CD11c or PerCP/Cy5.5 or PE/CY7 (Biolegend) (clone Bu15), CD45 FITC or PE/CY5 (BD Bioscience) (Clone HI30), Lineage (LIN) APC (Biolegend) (CD3/CD56/CD19/CD20, clones UCHT1; HIB19; 2H7; 5.1H11), CCR6 Brilliant Violet 711 (Biolegend) (Clone G034E3), CCR7 PE/DAZZLE (Biolegend) (Clone G043H7), CXCR3 Brilliant Violet 650 (Biolegend) (Clone G025H7), CXCR4 PE/CY5 (Biolegend) (Clone 12G5), CXCR5 Brilliant Violet 786 (Biolegend) (Clone J252D4) antibodies. Chemokines receptor expression was identified by comparison with an FMO (Fluorescence Minus One Control). Cells were gated as shown in **Supplementary Figure 1A**. After exclusion of doublets and dead cells, cells were gated for leukocytes (CD45^+^), T cells, B cells and NK cells were excluded [LIN^-^(CD19/CD20/CD56/CD3)]. Cells were then gated for myeloid dendritic cells with HLADR/CD11c^+^ gating; to identify the dendritic cell subset deriving from monocytes, cells were gated as CD14^h^/CD11^h^; finally, CD14 cells expressing CD209 marker were classified as CD209/CD14^+^ DC. The positive cells were gated against their FMO.

Samples were acquired using the Canto or Fortessa LSR II Flow Cytometers (Beckman Coulter and BD respectively) and analysed using Flowjo software (Treestar Inc.). To examine co-expression patterns of markers, the supervised algorithm SPICE or Simplified Presentation of Incredibly Complex Evaluations (Version 5.1) was used. SPICE is a data-mining software which enables the analysis of large data sets from polychromatic flow cytometry and organizes the normalized data graphically. A Boolean gating strategy was adopted to identify all possible cell populations and imported into the SPICE program. Pie charts generated as a result of this analysis represent frequency of co-expression of costimulatory markers whereby single costimulatory marker positive cells are represented by individual arcs surrounding the pie charts, while double, triple and quadruple costimulatory marker positive cells were represented by overlapping arcs (15, 33).

### PBMC Stimulation for Cytokine Intracellular Staining

PBMC were isolated and plated at the density of 1x10^6^/mL and rested overnight. The following day cells were either left untreated or stimulated with 100ng/ml LPS (Enzo Life Sciences), 3µM CPG ODN 2216 (*In vivo*gen) or 10µg/mL Poly (I:C) (*In vivo*gen) for three hours in the presence of 1X Brefeldin A and 1X Monensin (Biolegend). Intracellular cytokines IL-1β FITC (Clone JK1B-1), IL-12p40 Pacific Blue (Clone C11.5), IL-6 PE/DAZZLE (Clone MQ2-13A5), TNFα PE/CY7 (Clone MAb11) (all from Biolegend) were subsequently stained using the Fix/Perm kit (Life Technologies) according to manufacturer’s instructions. Samples were acquired using the Fortessa Flow Cytometer (Beckman Coulter) and analyzed using Flowjo software (Treestar Inc.).

### Antigen Uptake

Antigen uptake was evaluated by flow cytometry analysis as previously described (23, 34). Briefly, PBMC and synovial tissue were isolated/digested and washed in phosphate-buffered saline (PBS). The resulting single cell suspension was re-suspended in complete medium and transferred to flow-cytometry tubes containing 4 µl DQ™ Ovalbumin (DQ OVA, Molecular Probes). Tubes were incubated in parallel at 4 and 37°C for 15 mins and washed twice in cold FACS buffer. Cells were subsequently blocked with a human FcγR-binding inhibitor prior to staining, as described above. The incorporated fluorescence of the fluorescent reporters DQ-OVA (receptor-mediated endocytosis) was analysed by flow cytometry in the CD209/CD14^+^ gated cells, by following the excitation with the 488 nm laser and fluorescence using the 530/30 bandpass filter. The frequency of cells incorporating the DQ-OVA was calculated by subtracting cells incubated at 37°C (specific uptake) with cells incubated at 4°C (non-specific uptake).

### CD209^+^ DC Isolation and Expansion

We developed a novel protocol for magnetic isolation and expansion of the rare CD209^+^ DC population (described in **Figure 3A**). Following PBMC isolation, depletion of CD3/CD19/CD56 cells were obtained by magnetic positive isolation with the APC MicroBeads (Miltenyi Biotec) following manufacturer’s instructions. To spike the CD209 DC population, the lineage negative population was plated in a 6-well plate in the presence of 70 ng/mL granulocyte-macrophage colony stimulating factor (GM-CSF) (PeproTech) and 50 ng/mL interleukin-4 (IL-4) (PeproTech) and for 48 hours. CD209^+^ DC were then positively sorted by magnetic sorting using CD209 MicroBeads (Miltenyi Biotec) and used for further downstream analysis. Purity was assessed by flow cytometry. To test the effect of the synovial microenvironment on the CD209^+^ DC activation, cells were isolated from HC and plated at the density of 1x10^6^/mL for both flow cytometry and RT-PCR. After resting the cells for 1h, 20% synovial fluid (SF) from RA (n=5) or PsA (n=6) were added (or Media as control) for 18h. A similar protocol was utilized to stimulate the lineage negative cells in IA SF (n=4).

### Quantitative RT–PCR (qRT–PCR)

Total RNA was extracted using a miRNeasy Kit (Qiagen, Valencia, CA, USA). For mRNA analysis, cDNA was synthesized with a high‐capacity cDNA reverse transcription kit (Applied Biosystems). qRT–PCR was performed with SYBR green qPCR master mix (Applied Biosystems, Foster City, CA, USA) with specific primers, listed in **Table 2**. The relative level of each target transcript was calculated using the 2^–ΔΔCt^ method, relative to the basal. For HC, PsA and RA transcriptome comparison, 2^–ΔΔCt^ method, relative to the HC was performed. Heatmap was generated on -ΔCt values.

**Table 2:** Primers used for qPCR analysis in this study.

Gene name	FOR (5' 3')	REV (5' 3')
CCL2/MCP-1	GCTCGCTCAGCCAGATGCAA	1GG1GAAGTTI\TAACAGCAGGTGA
CXCL10	TICAAGGAGTACCTCTCTCTAGAA	GGT1GAITACTAATGCTGATGCAG
CXCL11	GGCTTCCCCATGTTCAAAAGAG	TCICAATATCTGCCACTITCACTG
RPLPO	GCGTCCTCGTGGAAG1GACATCG	ICAGGGAITGCCACGCAGGG
HPRT1	ATGGACAGGACTGAACGTCTTG	GGCTACAATGTGATGGCCTC
MMP9	TGT ACC GCT ATG GTTACA CIC G	GGC AGG GAC AGT TGC TIC T
MMP2	TAC AGG ATC AIT GGC TAC ACA C	GGTCAC AIC GCTCCA GAC T
CLU	GAG CGC AAG ACA C1G CTC A	TIC CCTGGTCICATTTAGGGC
SNX1	AAG CAC TCT CAG AATGGC TIC	CGG CCC TCC GTTTTTCAA G
SNX2	CCA CCC TAG AGTCAA GTC CATC	GAA ACT ICT TCT GTG GCTTCT GC
LAMP1	TCTCAG TGA ACT ACG ACA CCA	AGTGTA TGTCCTCTTCCAAAAGC
TLR4	AGA CCTGTC CCTGAACCC TAT	CGA TGG ACT TCT AAA CCA GCC A
TREM1	GAA CTC CGA GCTGCA ACTAAA	ICTAGC GTG TAG TCA CATTIC AC
MMP14	GGC TAC AGC AAT ATG GCT ACC	GAT GGC CGC TGA GAG TGA C
NOX2	TGTTCA GCTATG AGG TGG TGA	ICA GATTGG TGG CGTTAT TG

### Autologous Co-Culture

CD209^+^ DC were isolated from synovial fluid and left untreated or treated overnight with LPS (100ng/ml-Enzo Life Science). Autologous synovial CD4^+^ T cells were isolated from the same patient using the CD4 MicroBeads (Miltenyi Biotec) as per manufacturer’s instructions. T cells and CD209^+^ DC were co-cultured at a ratio of 10:1 for 3 days, after which sups were collected and cells were re-stimulated with the Cell Stimulation Cocktail (Containing PMA and Ionomycin, Biosciences) in the presence of Brefeldin A and Monensin (1X; Biolegend) for 5hr at 37°C. Cells were stained extracellularly for CD4 Pe/Cy7 (clone A161A1) and CD3 BV510 (clone OKT3) in addition to Live/Dead Infra-Red viability dye. Intracellular cytokine TNFα PerCP/Cyanine5.5 (clone Mab11), IFNγ APC (Clone 4S.B3), IL-2 BV605 (Clone MQ1-17H12), GM-CSF PE/Dazzle (Clone BVD2-21C11) were stained with the Fix/Perm kit (Life Technologies) according to manufacturer’s instructions. Samples were acquired using the Fortessa Flow Cytometer (Beckman Coulter) and analyzed using Flowjo software (Treestar Inc.).

### U-Plex MSD Assay

Isolated CD209^+^ DC were stimulated overnight with Tofacitinib 1µM or DMSO (as control) for 24 hrs. Supernatants were harvested and IL-1β, IL-6, IL-8 and IL-13 were analyzed by MSD assay (Meso Scale Diagnostics, USA) according to the manufacturer’s protocol. Data were normalized as expression relative to control (DMSO).

To identify possible differences in the synovial fluid of RA, PsA and OA patients, a customised MSD U-plex assay was utilised to simultaneously analyse the expression of chemokines (I-TAC, IP-10, MDC, SDF-1α, MCP-1, MCP-2, MCP-3, MCP-4, MIP-1α, MIP-1β, MIP- 3α, MIP-3β) and cytokines (GM-CSF, IL-12p70, IL-1β, IL-33, IL-4, TNFα, TNFβ).

### Migration Assay

Migration was assessed in a 24-trans-well plate with a 5 µm pore size (Sigma-Aldrich) as previously described (33). Isolated CD209^+^ DC from PBMC were stimulated overnight with 1µM Tofacitinib or DMSO (as control), cells were detached, centrifuged, and re-suspended in serum-free media. Cells were added to the top of the chamber at a concentration of 200,000 cells/mL. 2% FBS or 20% supernatant from paired SFMC (plated overnight) in serum-free media were added to the bottom of the chamber as chemo-attractants. Cells were incubated at 37°C for 1 h. Supernatants from the top and bottom of the chamber were collected in separate tubes and centrifuged. The pellets were re-suspended in 1% paraformaldehyde (PFA) for 10 min and washed with PBS. Cells were re-suspended in PBS, and 100 µL of Precision Counting Beads (BioLegend, USA) were added directly before flow cytometry analysis. Samples were acquired using the Fortessa Flow Cytometer (Beckman Coulter, USA) and analysed using FlowJo software (Treestar Inc., USA). Absolute cell counts were calculated as per the manufacturer’s instructions. The frequency was calculated by the ratio of cells that migrated to the bottom of the chamber *vs* the total number of cells.

### Statistical Analysis

Data were analyzed with the GraphPad Prism 9 software. Differences between groups were analysed by the non-parametric One-way analysis of variance (ANOVA) test, two-way ANOVA with Tukey multiple comparison or non-parametric t-test *p < 0.05, **p < 0.01, ***p < 0.001, ****p < 0.0001 values were considered as significant. Ordinary One-Way ANOVA was used in **Figure 4A**, following Shapiro Normality test. The test used can be found detailed in corresponding figure legends.

## Results

### Circulating CD209/CD14^+^ DC From IA Patients Display a Unique Pro-Inflammatory Phenotype

**Figure 1:**
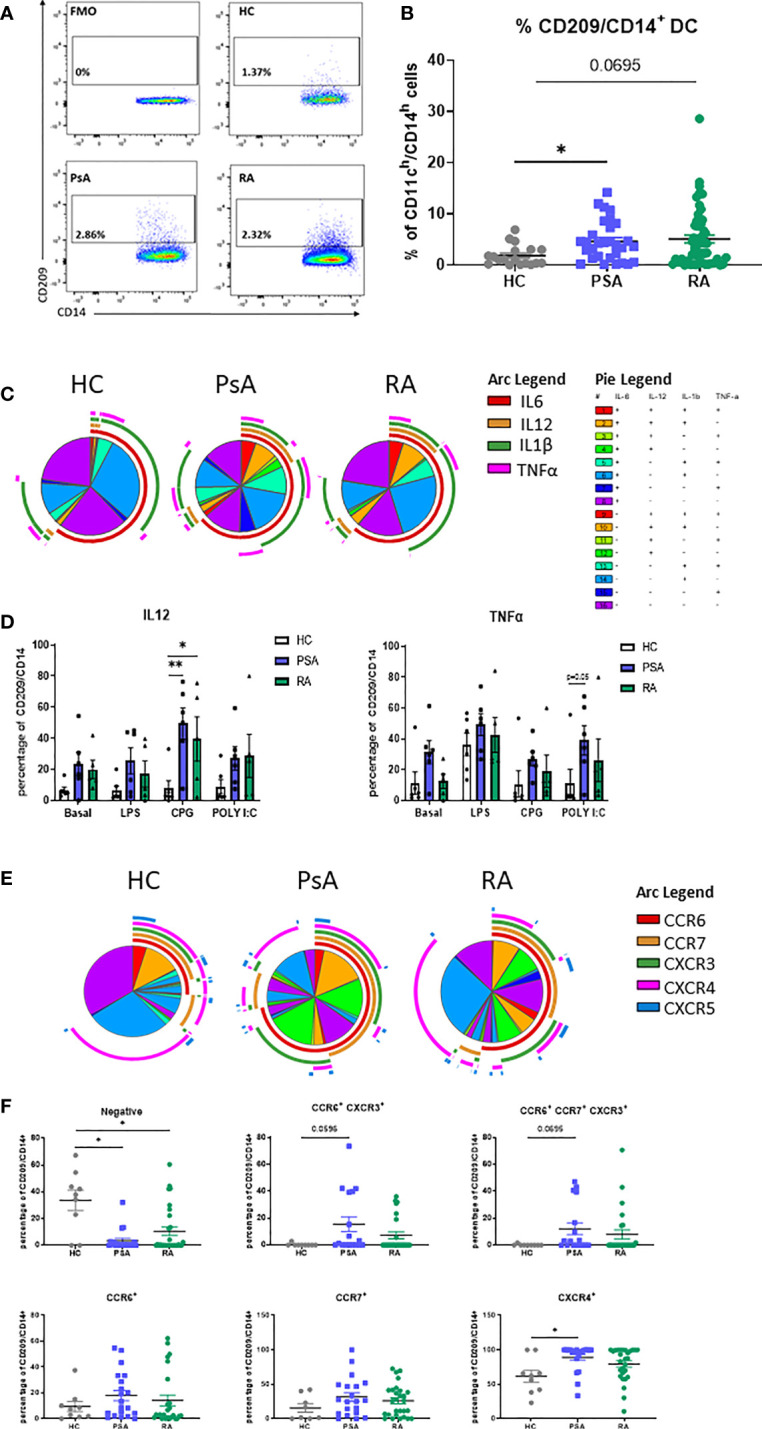
Circulating CD209/CD14^+^ DC and PsA patients display an active profile. PBMC from HC, PsA and RA patient were isolated and gated as per **Supplementary Figure 1B**. **(A)** Representative flow plot of FMO, HC, PsA and RA PBMC gated for circulating CD209/CD14^+^ DC. **(B)** Representative dot-plot showing the frequency of CD209/CD14^+^ DC, showing the frequency of the parent population (CD11c^h^/CD14^h^) in HC (n=19), PsA (n=30), RA (n=52) **(C)** SPICE algorithm flow cytometric analysis of unstimulated (basal) PBMC HC (n=6), PsA (n=6) and RA (n=5) CD209/CD14^+^ DC intracellularly stained for IL6 (red arc), IL12 (yellow arc), IL1β (green arc), TNFα (pink arc). **(D)** Representative histogram of PBMC cells left untreated (basal) or stimulated for 3 hours with LPS (100ng/ml) CPG ODN 2216 (3µM) or Poly (I:C) (10ug/mL) in the presence of Brefeldin A and Monensin. Data are represented as mean ± SEM and differences among groups were evaluated by two-way ANOVA with Tukey post-test. *p < 0.05, **p < 0.01. **(E)** SPICE algorithm flow cytometric analysis of peripheral blood HC (n=9), PsA (n=20) and RA (n=29) CD209/CD14^+^ DC expression displaying the frequency showing the frequency of the parent population (CD209/CD14^+^) of the chemokines receptors CCR6 (red arc), CCR7 (yellow arc), CXCR3 (green arc), CXCR4 (pink arc) and CXCR5 (blue arc) and corresponding **(F)** Dot plot of negative population (cells not expressing chemokine receptors), CCR6^+^CXCR3^+^, CCR6^+^CCR7^+^CXCR3^+^, CCR6^+^, CCR7^+^, CXCR4^+^ expressing cells. Data are represented as mean ± SEM and differences among groups were evaluated by non-parametric One-way ANOVA, Kruskal-Wallis test *p < 0.05.

In order to identify the CD209/CD14^+^ DC population derived from monocytes in the circulation, we isolated PBMC from HC, RA, PsA and OA patients. CD209/CD14^+^ DC were identified using the gating strategy shown in **Supplementary Figure 1A**. After exclusion of doublets and dead cells, cells were gated for leukocyte (CD45^+^), and T cells/B cells/NK cells excluded with a lineage marker antibody cocktail (CD19/CD20/CD56/CD3). Cells were then gated for myeloid DCs based on the expression of HLADR and CD11c. In order to identify the DC subset that were derived from monocytes, cells were gated as CD14^h^/CD11^h^; finally, CD14 cells expressing the CD209 marker were classified as CD209/CD14^+^ DC. The positive cells were gated against fluorochrome minus one (FMO) control. We identified, for the first time, the CD209/CD14^+^ DC in the circulation of HC, PsA and RA patients (**Figures 1A, B**), where we observed an increase in the % frequency of CD209/CD14^+^ DC in PsA (p<0.05) and RA patients (p=0.06) in comparison to HC. Interestingly, no differences were observed in the frequency of CD209/CD14^+^ DC between HC and OA patients (**Supplementary Figure 1B**). We next performed intracellular staining for IL-6, IL-12, IL-1β and TNF-α in untreated PBMC and PBMC treated with TLR ligand, as explained in material and methods. In untreated conditions we performed SPICE analysis, which allows the evaluation of single cytokine expression (single arc) and co-expressed cytokines (overlapping arcs). We observed that the frequency of CD209/CD14^+^ DC expressing and co-expressing the pro-inflammatory cytokines IL-6, IL-12, IL-1β and TNF-α were more complex in IA compared to HC, as evident by differential pie patterns and overlapping arcs (**Figure 1C**). In particular, we observed that PsA patients, at basal level, showed a differential co-expression of cytokines (for example IL-6 and TNFα- red and pink arcs), which was not observed in HC and RA. These differences were even more evident following stimulation with TLR ligands, where an increase in CD209/CD14^+^ DC expressing IL12 was observed in response to all TLR ligands in both RA and PsA *vs* HC, with a significant increase demonstrated for the TLR9 agonist CPG in PsA patients (p<0.05) and RA patients (p<0.01) (**Figure 1D**). In addition, we also observed increased frequency of TNFα in PsA in response to TLR3 agonist Poly I:C (p=0.05 PsA *vs* HC, **Figure 1D**). No effects were observed for IL-1β and IL-6 (data not shown).

The expression of chemokine receptors on DC are important for their migratory capacity but also elude to their maturation stage (35, 36). SPICE analysis clearly indicated that the expression and co-expression of CCR6, CCR7, CXCR3, CXCR4 and CXCR5 on the surface of CD209/CD14^+^ DC from PsA and RA patients, was more complex (overlapping of arcs and corresponding increase in pie segments) than that observed in HC (**Figure 1E** and **Supplementary Figure 2B** for the Pie legend). In particular, we observed a decrease in the percentage of cells not expressing chemokine receptors in PsA and RA *vs* HC (p<0.05). In addition, we observed an increase in the co-expression of certain receptors (CCR6/CXCR3 p=0.05 and CCR6/CCR7/CXCR3 p=0.06) in both PsA and RA *vs* HC. Furthermore, an increase in frequency of CD209/CD14^+^ DC singularly expressing CXCR4 was observed in PsA *vs* HC (**Figure 1F**, p<0.05). Interestingly, when we compared HC *vs* OA, we observed similar expression and co-expression patterns of chemokine receptors between the two groups (**Supplementary Figures 2A-C**), suggesting that the effect observed are specific to inflammatory arthritis. Overall, these data suggest that circulating IA CD209/CD14^+^ DC are more mature and possibly more migratory than that of HC.

### CD209/CD14^+^ DC Cells Accumulate Into the Joint of IA Patients

**Figure 2:**
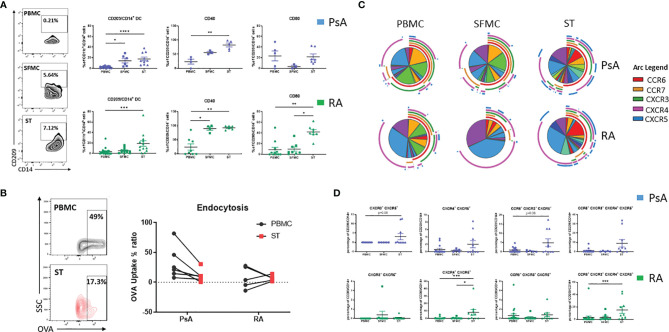
CD209/CD14^+^ DC are enriched in the joint of PsA and RA patients. **(A)** Left, representative flow plot of CD209/CD14^+^ DC frequency [showing the frequency of the parent population (CD11c^h^/CD14^h^)] in PBMC, SFMC and synovial tissue (ST). Right, Representative dot plots of frequency of CD209/CD14^+^ DC in PsA PBMC (n=14), SFMC (n=6), ST (n=10), CD40 expression PBMC (n=5), SFMC (n=3), ST (n=5), CD80 PBMC (n=6), SFMC (n=3), ST (n=7) and RA PBMC (n=21), SFMC (n=12), ST (n=12), CD40 expression PBMC (n=9), SFMC (n=4), ST (n=6), CD80 PBMC (n=12), SFMC (n=8), ST (n=8). Data are represented as mean ± SEM [frequency of the parent population (CD209/CD14^+^)] and Differences among groups were evaluated by non-parametric One-way ANOVA, Kruskal-Wallis test *p < 0.05. **p < 0.01, ***p < 0.001, ****p < 0.0001. **(B)** Endocytosis was evaluated in parallel at 4 and 37°C following antigen incubation. Left Representative flow plot of Receptor-dependent endocytosis (with OVA-DQ) in PBMC and ST. Right, Trending line of OVA-DQ update (expressed as ration between 37 and 4°C) in paired PBMC and ST (PsA n=6 and RA n=5). **(C)** SPICE algorithm flow cytometric analysis of PBMC (PsA n=14, RA n=21), SFMC (PsA n=5, RA n=9) and ST (PsA n=10, RA n=10) CD209/CD14^+^ DC displaying the frequency of the chemokines receptors CCR6 (red arc), CCR7 (yellow arc), CXCR3 (green arc), CXCR4 (pink arc) and CXCR5 (blue arc) and corresponding **(D)** Dot plot of different chemokine receptors co-expression. Data are represented as mean ± SEM and differences among groups were evaluated by non-parametric One-way ANOVA, Kruskal-Wallis test *p < 0.05. **p < 0.01, ***p < 0.001.

In order to assess the contribution of the CD209/CD14^+^ DC in synovial pathology, their characteristics at the site of inflammation was next examined. We assessed the frequency of CD209/CD14^+^ DC in the periphery *vs* the site of inflammation of IA patients, in particular in SFMC and synovial tissue (ST). Interestingly, we observed a stepwise increase in the frequency of CD209/CD14^+^ DC in SFMC and ST compared to the periphery in PsA patients (**Figure 2A** top; p<0.05 SFMC *vs* PBMC and p<0.0001 ST *vs* PBMC). This was also observed in RA patients for ST *vs* PBMC (**Figure 2A** bottom; p<0.001 *vs* PBMC). In addition, we observed a significant increase in CD209/CD14^+^ DC expression of the activation marker CD40 in PsA (p<0.01 ST *vs* PBMC), and RA (**Figure 2A** top; p<0.05 SFMC *vs* PBMC and p<0.01 ST *vs* PBMC) as well as CD80 in RA patients (**Figure 2A** bottom; p<0.001 ST *vs* PBMC and p<0.05 ST *vs* SFMC). These data suggest that once CD209/CD14^+^ DC migrate from the circulation to the joint they are further activated in response to microenvironmental cues in the inflamed joint. DC have high endocytic activity in their immature stage, which decreases with maturation (37), so we next evaluated the endocytic capacity of the CD209/CD14^+^ DC in PBMC and ST. As shown in **Figure 2B**, the CD209/CD14^+^ DC endocytic activity was lower in PsA ST compared to PBMC. Interestingly, a lack of endocytic activity was observed in RA CD209/CD14^+^ DC both in the periphery and ST, which is in agreement with our previous study which demonstrated reduced endocytic capacity of circulatory RA Mo-DC, an effect that was associated with an increased rate of differentiation (23). To further investigate the functional properties of the CD209/CD14^+^ DC, autologous co-culture between synovial CD4^+^ T cells and synovial CD209^+^ DC cells was performed, and intracellular staining was carried out 3 days after the co-culture. CD4^+^ T cells alone were used as control. Interestingly, an increase in GM-CSF, IL-2, TNFα and IFNγ was observed in CD4^+^ T cells when co-cultured with CD209^+^ DC cells (**Supplementary Figure 3**), suggesting CD209/CD14^+^ DC activate the production of pro-inflammatory cytokines by T cells. Cytokine expression was further increased by LPS treatment, suggesting the DC in the joint are susceptible to further activation by stimuli. Due to the rarity of the cells and the limit in the amount of fluid collected during arthroscopy only two samples were performed, further studies are needed to expand and confirm these observations.

We next evaluated the chemokine receptor expression CCR6, CCR7, CXCR3, CXCR4 and CXCR5 in CD209/CD14^+^ DC in the periphery *vs* the site of inflammation in both PsA and RA. A complex expression and co-expression of chemokine receptors at the site of inflammation was observed which was particularly evident for both PsA and RA ST (**Figure 2C**). In particular, we observed an increase in the co-expression of CXCR3/CXCR5 in PsA (p=0.08 ST *vs* PBMC, **Figure 2D**) and a significant increase in CXCR4/CXCR5 RA (p<0.001 ST *vs* PBMC and p<0.05 ST *vs* SFMC). The co-expression of CCR6/CXCR3/CXCR5 was higher in ST of PsA patients (p=0.06 *vs* PBMC) and the co-expression of CCR6/CXCR4/CXCR4/CXCR5 was significantly higher in ST of RA patients (p< 0.001 *vs* PBMC). These data suggest that IA CD209/CD14^+^ DC are further activated at the site of inflammation, with differential chemokine receptor expression patterns demonstrated between PsA and RA.

### Isolation/Expansion and Transcriptional Characterization of Novel CD209/CD14^+^ DC

**Figure 3:**
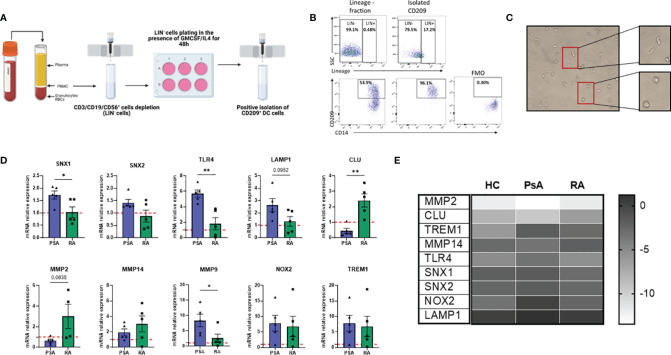
New protocol for CD209^+^ DC isolation/expansion and transcriptional analysis. **(A)** Diagram of steps involved in CD209^+^ DC isolation (Created with BioRender.com). PBMC were isolated from blood and CD3/CD19/CD56 (Lineage, LIN) cells were depleted by positive isolation. The LIN^-^ cells were plated and treated with a cocktail of 70 ng/mL (GMCSF) (PeproTech) and 50 ng/mL IL-4 (PeproTech) to spike the CD209 population. 48 hours after CD209^+^ DC were sorted by positive magnetic isolation. **(B)** Flow plot to assess the purity of the CD209^+^ cells. The frequency of CD209 cells was around 50%, which was enriched to >96% in the isolated CD209^+^ cells were positive. FMO gating is also shown. **(C)** Representative microscopic images (Leica inverted microscope 40X) showing the morphology of isolated CD209^+^ DC. Cells appear heterogeneous with some more round shape cells and some elongated cells (in the magnification). **(D)** qPCR displaying the relative gene expression of *MMP9, MMP2, CLU, SNX1, SNX2, LAMP1, TLR4, TREM1, MMP14, NOX2* in isolated CD209^+^ DC from HC (n=5), PsA (n=5) and RA (n=5) patients. Data are expressed as relative expression to HC (represented as red dotted line) and was calculated with the 2^–ΔΔCt^ method. Data are represented as mean ± SEM and differences among groups were evaluated by Non-parametric One-way ANOVA, Kruskal-Wallis test *p < 0.05, **p < 0.01”. **(E)** Heatmap representing -ΔCt value from HC (n=5), PsA (n=5) and RA (n=5) RNA isolated from circulatory CD209^+^ DC.

Isolation of rare population of dendritic cells is technically challenging. Herein, we developed a protocol to isolate this rare DC population by magnetic cell sorting by altering the protocol previously described by Engering and colleagues (38). After PBMC isolation, cells were magnetically sorted, depleting T/B and NK cells (with the use of CD3/CD19/CD56 APC markers) (**Figure 3A**). The lineage negative population was plated in a 6-well plate in the presence of GM-CSF and IL-4 cytokines (classically used for Mo-DC differentiation) for 48 hours. The enriched CD209^+^ cells were then positively isolated by magnetic sorting and used for further downstream analysis. The purity obtained with this protocol was > 96% (**Figure 3B**) and the cells displayed a heterogeneous phenotype and morphological characteristics, with a mixture of elongated cells and round morphological appearance (**Figure 3C**), suggesting CD209/CD14^+^ DC in the circulation are at various stages of differentiation/maturation.

In order to investigate possible transcriptional differences between CD209/CD14^+^ DC from PsA and RA patients, we isolated circulatory CD209^+^ cells from HC, PsA and RA patients and performed RNA extraction and qPCR. Interestingly, genes involved in endocytosis/antigen processing (*SNX1, SNX2, TLR4, LAMP1*) (39–42) were all found to be higher in PsA, compared to RA (**Figure 3D**) which is in agreement with the impaired endocytosis observed in the RA patients (**Figure 2B**). In contrast, clusterin (*CLU*), a gene involved in efferocytosis (clearing of dead cells) (43) was found to be higher in RA CD209^+^ DC compared to PsA. We also found an inverse relationship between *CLU* and *MMP9* expression which is consistent with previous studies (44). In contrast, *MMP2* (p=0.06), and to a lesser extent MMP14, were found to be higher in RA patients compared to PsA. No differences in the expression of NOX2 and TREM1 were observed between RA and PsA (**Figure 3D**). Heatmap analysis compared the inverse ΔCt (-ΔCt) of the genes of interest between the 3 groups (HC, PsA and RA), underlining differences in gene expression among the groups (**Figure 3E**).

### Effect of Joint Micro-Environment on CD209/CD14^+^ DC Activation

**Figure 4:**
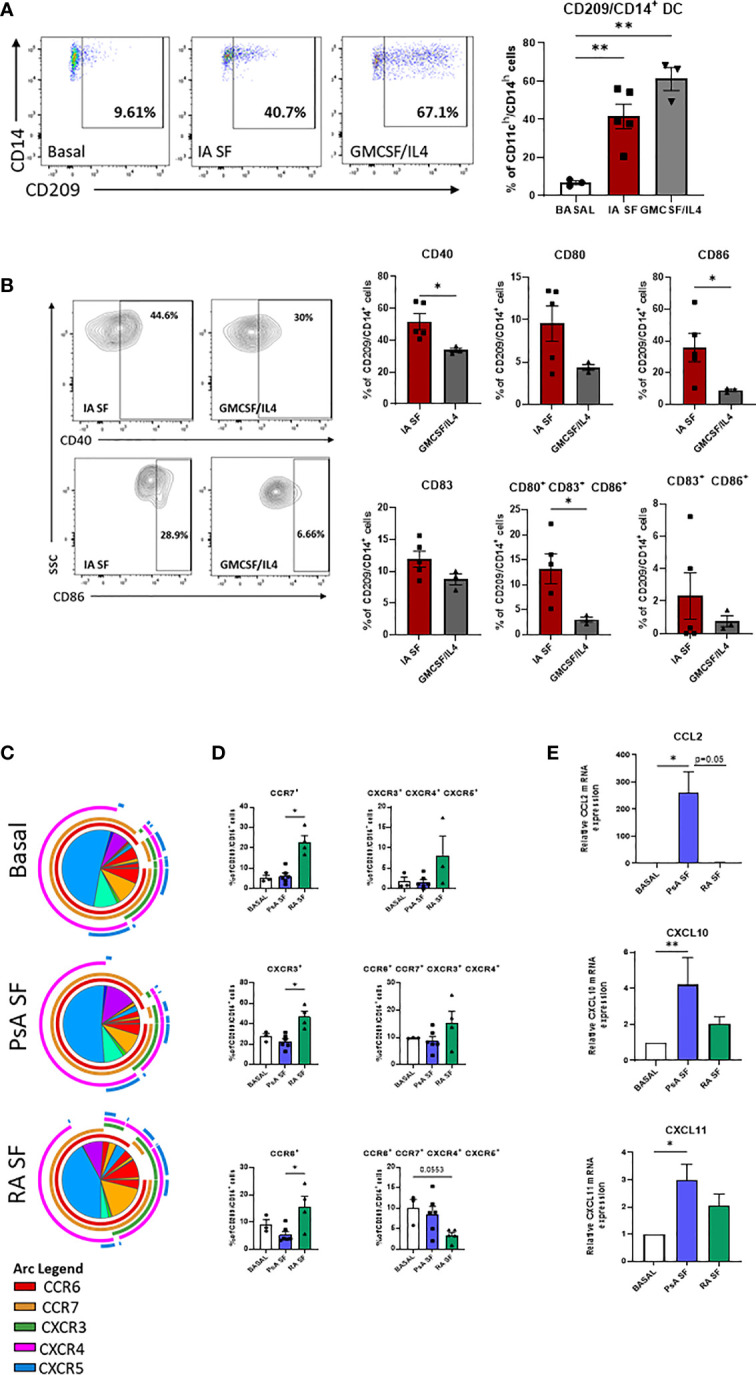
RA and PsA micro-environment modulate CD209/CD14^+^ DC development and activation. **(A)** Left, Representative flow plot of lineage negative cells from HC (n=2) left untreated (Basal n=3), treated with 20% IA synovial fluid (SF n=4), or with GMCSF/IL4 (n=3) cocktail. Right, histogram representing the frequency of CD209/CD14^+^ DC. Data are represented as mean ± SEM and differences among groups were evaluated by non-parametric One-way ANOVA. **p < 0.01. **(B)** histograms representing frequency of the maturation markers CD40, CD80, CD83, CD86 singularly expressed or combinations. Data are represented as mean ± SEM and differences among groups were evaluated by non-parametric t-test (Mann-Whitney test) *p < 0.05. **(C)** SPICE algorithm flow cytometric analysis of isolated CD209^+^ DC from HC left untreated (n=3) or stimulated with 20% SF from PsA (n=6) and RA (n=4), displaying the frequency of the chemokines receptors CCR6 (red arc), CCR7 (yellow arc), CXCR3 (green arc), CXCR4 (pink arc) and CXCR5 (blue arc) and **(D)** corresponding histograms representing the expression and co-expression of the chemokine receptors. Data are represented as mean ± SEM and differences among groups were evaluated by Non-parametric One-way ANOVA, Kruskal-Wallis test *p < 0.05. **(E)** qPCR displaying the relative gene expression of *CCL2*, *CXCL10* and *CXCL11* in isolated CD209^+^ DC from HC left untreated (n=4) or stimulated with 20% SF from PsA (n=5) and RA (n=5). Relative expression to basal was calculated with the 2^–ΔΔCt^ method. Data are represented as mean ± SEM and differences among groups were evaluated by Non-parametric One-way ANOVA, Kruskal-Wallis test *p < 0.05, **p < 0.01.

To evaluate the effect of the joint micro-environment on the specific DC subset development the lineage negative population derived from HC peripheral blood was left untreated or treated with 20% synovial fluid (SF, PsA and RA) or with the cytokine cocktail (GM-CSF/IL-4). As expected, the frequency of CD209/CD14^+^ DC cells were significantly increased in response to the GMCSF/IL-4 cytokine cocktail (p<0.001 **Figure 4A**), interestingly, we also observed a strong increase in the frequency of CD209/CD14^+^ DC following treatment with IA SF (p<0.001) (**Figure 4A**), thus suggesting that the SF contains soluble mediators that can induce the development of the CD209/CD14^+^ DC subset. In contrast, synovial fluid from OA patients had no effect on the frequency of the DC subset (**Supplementary Figures 4A, B**).

Analysis of the maturation stage of CD209/CD14^+^ DC in response to IA SF demonstrated that the joint microenvironment is not only capable of boosting the development of CD209/CD14^+^ DC, but it also drives a more mature and poly-mature phenotype, as observed by the increase in the co-expression of maturation markers when compared to GM-CSF/IL-4 stimuli. In particular, IA SF led to a significant increase of CD40 (p<0.05) and CD86 (p<0.05) and CD80/CD83/CD86 co-expression (p<0.05) (**Figure 4B**). No differences were observed between RA and PsA SF. In contrast OA SF failed to trigger the development of the DC subset (**Supplementary Figure 4B**).

We then sought to investigate the effect of the joint micro-environment on the CD209/CD14^+^ DC chemokine receptor expression, CD209^+^ cells were isolated and stimulated overnight with 20% SF from both PsA and RA patients (**Figures 4C, D**). SPICE analysis indicated that RA SF drives a more complex chemokine receptor co-expression pattern than PsA in the CD209/CD14^+^ DC (**Figure 4C**). This was highlighted by an increase in CCR6, CCR7 (p<0.05), CXCR3 (p<0.05) and in the co-expression of CXCR3/4/5, whereas a decrease was observed in the co-expression of CCR6/CCR7/CXCR4/CXCR5 (**Figure 4B**). OA SF failed to activate the DC subset (**Supplementary Figure 4C**). To further investigate the ability of the CD209^+^ DC to recruit other immune cells when subjected to the joint micro-environment, we evaluated the mRNA expression of CCL2 (MIP-1), CXCL10 and CXCL11, known chemoattractants for immune cells, especially monocytes and T cells (45–47). All three genes were modulated by both PsA and RA SF, with a significant increase only observed for PsA SF (CCL2 p<0.05, CXCL10 p<0.01, CXCL11 p<0.05) (**Figure 4E**). Multiplex analysis of SF demonstrated that all chemokines tested were higher in RA SF *vs* PsA SF (**Supplementary Figures 5A, B**, all p<0.05), including MCP-1/4, IP-10, MIP-1α/β, MIP-3α, suggesting that the increase in these soluble mediators in RA SF may in part be responsible for the observed increase in chemokine receptor expression on CD209^+^ DC cells. As expected, IL-12p70 was found to be higher in PsA *vs* RA SF (**Supplementary Figures 5A, B**), while IL-1 β was higher in RA *vs* PsA SF. Interestingly minimum expression of all cytokines/chemokines were observed in OA SF, thus possibly explaining the lack of effect of OA SF on modulation of CD209^+^ DC activity (**Supplementary Figure 4C**).

### Therapeutic Targeting of CD209/CD14^+^ DC

**Figure 5:**
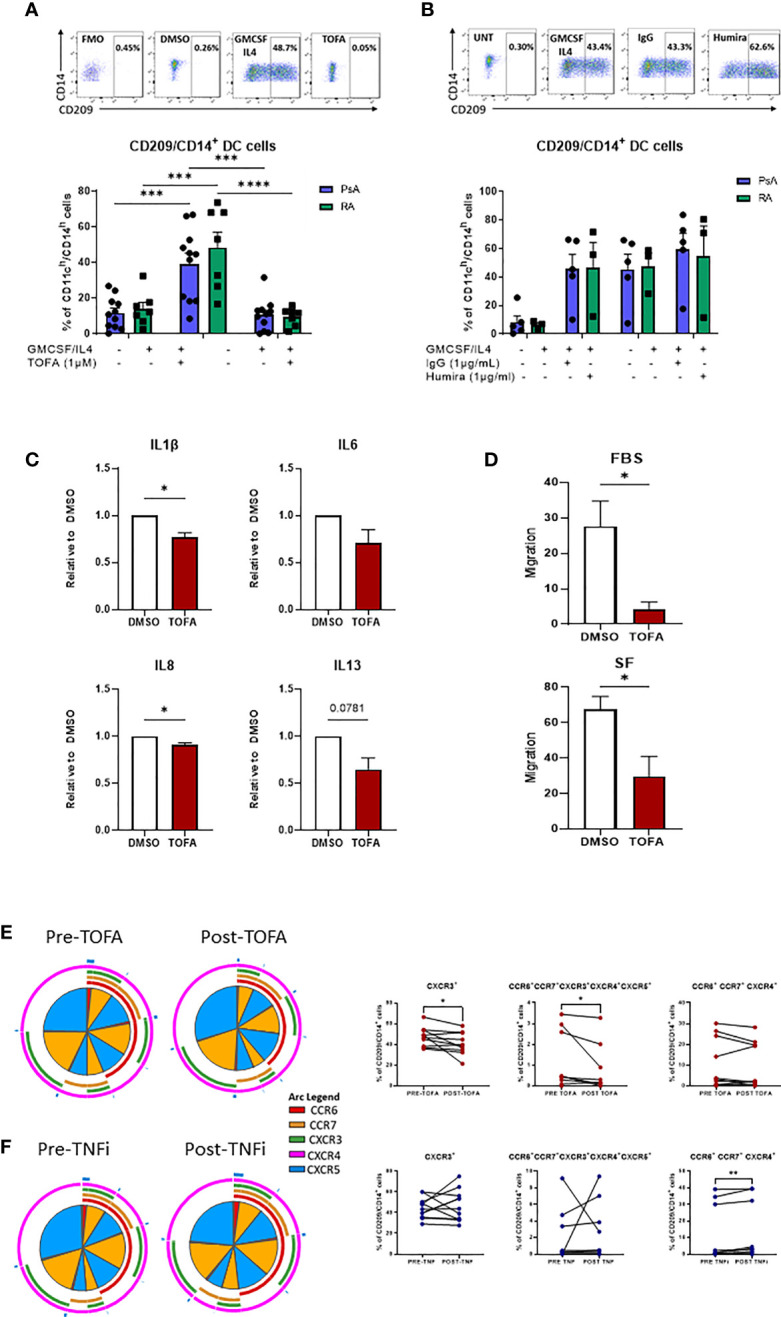
Therapeutic targeting the CD209/CD14^+^ DC population. **(A)** Lineage negative population from PsA (n=11) and RA (n=7) patients treated 15’ with DMSO/Tofacitinib (1uM) prior to the stimulation with the GMCSF/IL4 cocktail. Top, representative flow plot; bottom histograms. **(B)** Lineage negative population from PsA (n=5) and RA (n=3) patients treated 15’ with IgG/Humira (1ug/mL) prior to the stimulation with the GMCSF/IL4 cocktail. Top, representative flow plot; bottom histograms. Data are represented as mean ± SEM and differences among groups were evaluated by two-way ANOVA with Tukey post-test. *p < 0.05, **p < 0.01, ***p < 0.001, ****p < 0.0001. **(C)** Multiplex MSD analysis of IL1β, IL6, IL8, IL13 from CD209^+^ DC supernatant of IA (n=7) patients treated over-night with DMSO/Tofacitinib (1µM). Data are represented as ratio of expression toward control (DMSO) ± SEM and differences among groups were evaluated by paired t-test *p < 0.05, **p < 0.01. **(D)** Migration analysis of CD209^+^ DC from IA (n=3) patients treated over-night with DMSO/Tofacitinib (1µM). Histograms display the migration ratio toward 2% FBS or 20% SFMC paired supernatants, between the bottom of the well and the total cell count as described in the methods. differences among groups were evaluated by paired t test. *p < 0.05. E-F) PBMC from patients before and after **(E)** Tofacitinib treatment (n=10) or **(F)** TNF inhibitor (TNFi) treatment (n=11). Left, SPICE algorithm flow cytometric analysis displaying the frequency of the chemokines receptors (shown as frequency of parent) CCR6 (red arc), CCR7 (yellow arc), CXCR3 (green arc), CXCR4 (light blue arc) and CXCR5 (dark blue arc) and right, corresponding trending lines for chemokine receptors expressing alone or in combination. Differences among groups were evaluated by paired t test.

Our previous study suggested that Mo-DC development could be targeted by inhibition of the JAK/STAT pathway (23), therefore, here we sought to investigate the effect of JAK/STAT inhibition in comparison to TNFi on CD209/CD14^+^ DC function. To investigate the effect of JAK/STAT inhibition on CD209/CD14^+^ DC development, CD3/CD19/CD56 cells were magnetically sorted from PBMC and the lineage negative cells stimulated with a GM-CSF/IL-4 cocktail in the presence or absence of DMSO/TOFA (1µM), or, as a comparison, in the presence or absence of IgG/Adalimumab (Humira) (1µg/mL). Interestingly, after 2 days of differentiation, Tofacitinib significantly decreased the frequency of the CD209/CD14^+^ DC in both PsA (p<0.001) and RA (p<0.0001) (**Figure 5A**). This effect was not observed in response to Adalimumab (**Figure 5B**).

Further analysis of the effect of Tofacitinib on isolated CD209^+^ DC demonstrated a significant decrease in IL-1β and IL-8 (p<0.01), IL-13 (p<0.05) and IL-6 (p=0.07), thus decreasing the inflammatory phenotype of CD209/CD14^+^ DC (**Figure 5C**).

To evaluate whether Tofacitinib could influence the migratory capacity of CD209^+^ DC to the joint, we performed a migration assay. Treatment of CD209^+^ DC with Tofacitinib significantly inhibited their migratory capacity towards a chemo-attractant (2% FBS) (p<0.05) (**Figure 5D**). Furthermore, when migration was examined in response to the patients paired synovial fluid, we also observed a significant decrease in migratory capacity (p<0.05). (**Figure 5D**).

Finally, we collected blood from 10 patients pre/post Tofacitinib and 11 patients pre/post TNFi treatments, to examine the effect on CD209/CD14^+^ DC activation *in vivo*. Notably, the frequency of CXCR3 expressing cells was significantly decreased after treatment with Tofacitinib (**Figures 4E, F** p<0.05), together with a reduction in the co-expression of all five chemokine receptors expression in response to Tofacitinib (**Figures 5E, F** red section of pies and overlapping of all arcs, p<0.05). These effects were not observed in patients following treatment with TNF inhibitors, also indeed a significant increase in CCR6^+^CCR7^+^ CXCR4^+^ expressing cells was observed (**Figures 5E, F**, p<0.05), thus suggesting CD209/CD14^+^ DC are sensitive to regulation by JAK/STAT, but not TNFi, treatments.

## Discussion

In this study, we identified a DC population that derive from monocytes, characterized as CD209/CD14^+^ DC. This CD209/CD14^+^ DC population is present in the circulation of HC, with increased frequency in RA and PsA patients. We demonstrate, for the first time, that circulatory IA CD209/CD14^+^ DC express more cytokines and display a unique chemokine receptor expression and co-expression profile compared to HC. In addition, we observed that CD209/CD14^+^ DC are enriched at the site of inflammation, where they undergo further activation and priming. We also describe a new protocol for the isolation and expansion of CD209^+^ DC and demonstrate that circulatory CD209/CD14^+^ DC exhibit differential gene expression in RA *vs* PSA. The joint micro-environment induces the development of these cells, in addition to their maturation status. We also observed a difference between the migratory capability of these cells in RA *vs* PsA, with RA cells expressing more chemokine receptors when challenged with synovial fluid, while more chemo-attractants were expressed following culture with PsA SF. We finally demonstrate that JAK/STAT inhibition, but not the TNFi, alters CD209/CD14^+^ DC development and activation, with Tofacitinib also inhibiting cytokine production and migratory capacity.

DC have been shown to have a key role in the initiation and progression of inflammatory arthritis, in both human and rodents (17, 18). The advance of new technology, including systems immunology and single-cell analysis, has aided scientists to identify new DC subsets in humans, therefore to date, five distinct DC subsets are known to be present in human: the pDC, the classical myeloid subsets cDC1 (CD141^+^) and cDC2 (CD1c^+^), the DC3 and the monocyte-derived DC population (11, 12). The CD141^+^ and the CD1c^+^ DC cells are in a semi-mature stage in circulation in IA patients and are enriched at the site of inflammation, where they are further activated (13–16). Here we show that the CD209/CD14^+^ DC are present in the circulation of HC, PsA and RA patients, with a higher frequency observed in RA and PsA patients, but not OA. Further analysis of activation markers demonstrated a higher level of IA DC expressing cytokines under basal conditions and in response to TLR stimulation compared to HC. TLR recognition in DCs is important for their response to foreign organisms, which initiate a series of events that link innate and adaptive immunity (48). In particular, we observed an increase in IL-12 in both PsA and RA DC in response to CPG (TLR9) stimuli and TNFα in PsA in response to TLR3 stimuli (Poly I:C); both cytokines having a key role in DC immuno-biology, conferring a more mature phenotype to DC, leading to T cells activation (49, 50). Different studies have underlined the importance of chemokine receptor expression in DC, and in particular, Mo-DC for maturation and trafficking (35, 36). Overall we observed a more active and mature CD209/CD14^+^ DC phenotype in the periphery of IA patients, when compared to HC, as underlined by the increase in individually expressed chemokine receptors, including CCR7, important for migration of DC to lymph node (51, 52), and CXCR4, which is known to be important in DC recruitment in a CIA model (53), together with the decrease in the frequency of cells lacking chemokine receptors. In addition, we observed an increase in cells co-expressing chemokine receptors together, including CCR6^+^CXCR3^+^ and CCR6^+^CCR7^+^CXCR3^+^ in DC cells from PsA *vs* HC. Due to the rarity of DCs subsets and lack of definitive markers, very few studies have identified DC subsets at the site of inflammation, the joint of IA patients, with most studies focused on the myeloid CD141 and CD1c cells (13–15). Here, we identified the CD209/CD14^+^ DC are enriched in both the PsA and RA joint, with a higher frequency of these cells observed in both SF and ST. Once CD209/CD14^+^ DC reach the joint, they are more mature, as observed by the increased expression of both CD40 and CD80 markers, paralleled by a loss of their endocytic activity. The increased maturation was observed in both RA and PsA patients, however, while endocytosis capacity decreased in the ST *vs* blood in PsA, in RA endocytic activity was already impaired in circulation which is supported by previous observations suggesting circulatory RA Mo-DC are already primed (23). While losing their endocytic activity CD209/CD14^+^ DC acquire antigen presenting properties, as evidenced by the observed increase in cytokine expression in synovial CD4^+^ T cells co-cultured with CD209^+^ DC. Due to the rarity of the cells and the limit in the amount of fluid collected at arthroscopy this was performed only on two samples, further studies are needed to expand and confirm these observations in a bigger cohort, and possibly to discriminate between RA and PsA CD209^+^ DC APC properties. On entering the site of inflammation, IA DC display a more complex expression and co-expression pattern of chemokine receptors, which are known to synergistically promote leukocyte migration (54). Although co-expression of chemokine receptors has not been widely studied in DC, it has been shown in rodents that autoimmune uveitis induced by interphotoreceptor retinoid binding protein (IRBP) is associated with DC migration of cells specifically co-expressing CXCR3 and CXCR5 (55), allowing us to hypothesize that CD209/CD14^+^ DC expressing multiple chemokine receptors together have higher migration capacity.

Optimization of a novel protocol for isolation and expansion of CD209^+^ DC from blood, allowed us to study, more meticulously, the CD209^+^ DC population ex-vivo, including their transcriptional and migratory capacity. In order to investigate possible transcriptional differences between CD209/CD14^+^ DC from PsA *vs* RA patients, we isolated circulatory CD209^+^ cells from HC, PsA and RA patients and performed qPCR. Genes involved in endocytosis/antigen processing (*SNX1, SNX2, TLR4, LAMP1*) (39–42) were all found to be higher in PsA, compared to RA, which is in agreement with the impaired endocytosis observed in the RA patients, and our previous observations in *in-vitro* generated Mo-DC (23). In contrast, clusterin (*CLU*), a gene involved in efferocytosis (clearing of dead cells) (43) was found to be higher in RA CD209^+^ DC compared to PsA. We also found an inverse relationship between *CLU* and *MMP9* expression which is consistent with previous studies (44). In contrast, MMP2 and to a lesser extent *MMP14*, were found to be higher in RA patients compared to PsA. No differences were observed in *NOX2* and *TREM1* expression between RA and PsA.

In our previous studies, we observed that circulatory RA monocyte were primed to differentiate to DC compared to PsA and HC monocytes (23), in addition RA monocytes stimulated with LPS express a distinct M1-like macrophage-gene signature (33). This suggests that circulating RA monocyte are primed to differentiate into monocytic-derived cells, with the differentiation route defined by environment cues within the inflamed joint. In this study we observed that synovial fluid was sufficient to induce differentiation to CD209/CD14^+^ DC, which was similar to that observed in response to the classical Mo-DC (GM-CSF/IL-4) cocktail stimulation. In addition, the IA micro-environment led to a multi-mature phenotype of the DC subset, with an increase of CD40, CD80 and CD86 singularly expressed, when compared to the GMCSF/IL4, and the increase of CD80/CD83/CD86 co-expressing cells. This suggests that the inflammatory milieu of the IA joint regulates DC differentiation and maturation of the CD209/CD14^+^ DC subset. This effect was specific to IA fluid; in fact, we did not observe any priming when cells were cultured with OA fluid. When CD209^+^ DC were exposed to RA SF we observed an increase in CCR7 and CXCR3, together with an increase in the co-expression of CXCR3/CXC5/CXCR5 and CCR6/CCR7/CXCR3/CXCR4 suggesting that the joint micro-environment enhances CD209/CD14^+^ DC trafficking. Furthermore PsA SF, and to a lesser extent RA SF, induced an increase in the chemokines CCL2, CXCL10 and CXCL11, suggesting a further recruitment of immune cells, in particular monocytes and T cells (45–47). Thus, we could speculate that the differential effects observed in response to the RA and PsA joint microenvironment may lead to differences in immune cell recruitment, including CD209/CD14^+^ DC to the site of inflammation. A more in-depth analysis of synovial fluid from RA, PsA and OA patients confirmed the presence of multiple chemoattractants in IA SF, as opposed to OA SF where minimal levels were observed. In addition, most of the chemokines were found to be higher in RA *vs* PsA fluid, which may in part explain the increase in chemokine receptor expression observed in DC cells exposed to RA, but not PsA SF.

Next, we examined the effect of current therapeutic targets on CD209/CD14^+^ DC development. Treatment with Tofacitinib resulted in a significant decrease in the frequency of CD209/CD14^+^ DC in both RA and PsA patients, suggesting that the JAK/STAT pathway is involved in the development of CD209/CD14^+^ DC subset, which is in agreement with our previous studies (23). No effect was observed with the TNFi. Furthermore, Tofacitinib treatment reduced the production of selective inflammatory cytokines in IA CD209^+^ DC, including IL-1β, IL-6, IL-8 and IL-13, thus reducing their inflammatory phenotype. Furthermore, we demonstrated that Tofacitinib significantly inhibited the migratory capacity of CD209^+^ DC, suggesting the JAK/STAT pathway may be involved in DC recruitment to the joint. Finally, in patients receiving the JAK/STAT inhibitor, we observed a decrease in the expression of chemokine receptors, including CXCR3 as well as in the co-expression of all five chemokine receptors’ expression and their combination on CD209/CD14^+^ DC, with minimal, if not opposite, effects observed for the patients treated with TNFi.

**Figure 6:**
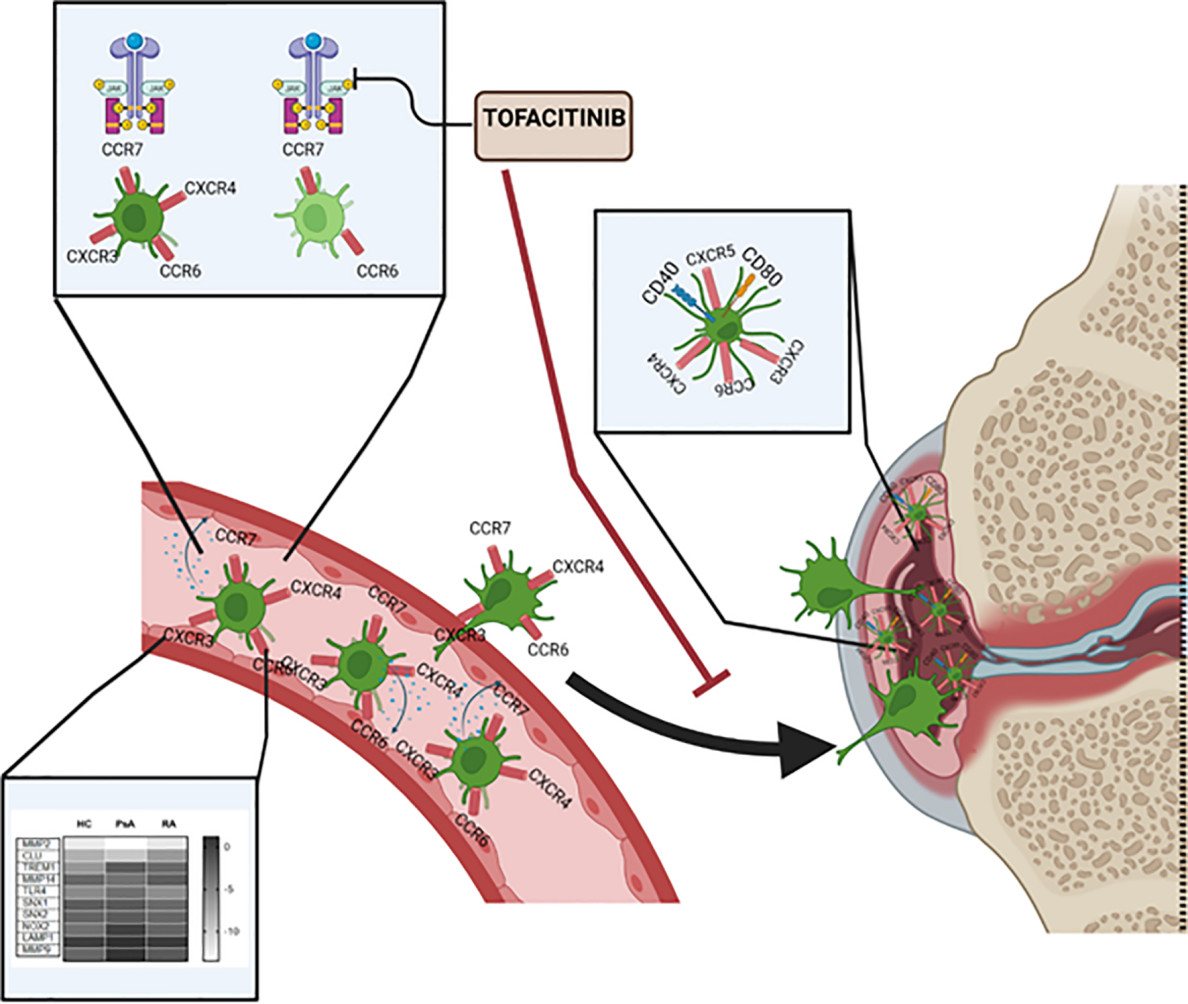
Graphical summary of key results described within the manuscript. CD209/CD14^+^ DC in the circulation of PsA and RA patients present increased frequency, cytokine production and chemokine receptors compared to healthy individual. The CD209/CD14^+^ DC are enriched in the IA joint where they are further activated, possibly by inflammatory mediators present in the fluid, and exhibit a unique mature and migratory phenotype, as observed by the increase in CD40 and CD80, and in chemokine receptors alone or in combinations. Key transcriptional differences in circulatory CD209^+^ DC from RA and PsA patients were observed, in particular in genes involved in endocytosis/antigen presentation. Tofacitinib specifically targets the development, functional capacity and migration of CD209/CD14^+^ DC subset.

In conclusion, as summarized in **Figure 6**, we have characterized a new subset of monocyte-derived dendritic cell CD209/CD14^+^ DC in the circulation of PsA and RA patients. We observe an increase in frequency, cytokine production and chemokine receptor expression of these cells in IA patients in respect to healthy individuals. The CD209/CD14^+^ DC are enriched in the IA joint where they are further activated and exhibit a unique mature and migratory phenotype, as observed by the increase in CD40 and CD80, and in the expression of chemokine receptors, alone or in combination. We optimized a novel protocol for the isolation and expansion of the CD209^+^ DC cells by magnetic sorting, which allowed us to investigate transcriptional differences in circulatory CD209^+^ DC from RA and PsA patients. Interestingly, we observed differences in genes involved in endocytosis/antigen presentation with RA and PsA patients. In addition, we observed that the joint micro-environment from IA, but not OA patients, increases the activation, maturation, and possible migratory capacity of these cells. Finally, we demonstrated that Tofacitinib specifically targets the development, functional capacity, and migration of CD209/CD14^+^ DC subsets. Taken together, these data identify a new DC subset in RA and PsA patients, which may contribute to joint inflammation.

## Data Availability Statement

The original contributions presented in the study are included in the article/**Supplementary Material**. Further inquiries can be directed to the corresponding author.

## Ethics Statement

The studies involving human participants were reviewed and approved by St. Vincent’s University Hospital and Tallaght Univeristy Hospital Ethics and Medical Research Committee and was performed in accordance with the Declaration of Helsinki. The patients/participants provided their written informed consent to participate in this study.

## Author Contributions

VM and UF designed the experiments. VM, MC, and AF performed experiments. KF helped in patient recruitment and processing. DV and RM recruited the patients. VM analyzed the data. VM and UF wrote the manuscript with all authors revising the manuscript. UF supervised the project. All authors contributed to the article and approved the submitted version.

## Funding

This work was funded by UCB Newman Fellowship.

## Conflict of Interest

The authors declare that the research was conducted in the absence of any commercial or financial relationships that could be construed as a potential conflict of interest.

## Publisher’s Note

All claims expressed in this article are solely those of the authors and do not necessarily represent those of their affiliated organizations, or those of the publisher, the editors and the reviewers. Any product that may be evaluated in this article, or claim that may be made by its manufacturer, is not guaranteed or endorsed by the publisher.

## Supplementary Material

Supplementary Figure 1
**(A)** Gating strategy for CD209/CD14^+^ DC. Cells were gated based on forward and side scatter and dead cells and doublets were removed. Live Dead Near-IR (Molecular Probes) was used to eliminate dead cells. The cells were then gated as CD45^+^/LIN^−^(CD19-CD20-CD56-CD3)/CD11c^+^/HLADR^+^ CD14^h^/CD11c^h^ CD209^+^/CD14^+^. The positive CD209/CD14^+^ DC population was characterized against the FMO (Fluorescence Minus One Control). **(B)** Representative dot-plot of frequency of CD209/CD14^+^ DC, showing the frequency of the parent population (CD11c^h^/CD14^h^) in HC (n=19) and OA (n=6).

Supplementary Figure 2
**(A)** SPICE algorithm flow cytometric analysis of peripheral blood HC (n=9) and OA (n=6). CD209/CD14^+^ DC expression showing the frequency (represented as frequency of the parent population (CD209/CD14^+^)) of the chemokines receptors CCR6 (red arc), CCR7 (yellow arc), CXCR3 (green arc), CXCR4 (pink arc) and CXCR5 (blue arc) and corresponding **(B)** Pie Legend for all the combination of chemokine receptors. **(C)** Dot plot of negative population (cells not expressing chemokine receptors), CCR6^+^CXCR3^+^, CCR6^+^CCR7^+^CXCR3^+^, CCR6^+^, CCR7^+^, CXCR4^+^ expressing cells. Data are represented as mean ± SEM

Supplementary Figure 3 Intracellular staining for GM-CSF, IL-2, IFN γ and TNFα expressed in synovial CD4^+^ T cells alone or co-cultured with synovial CD209^+^ DC. Left, representative flow plot. Right, Line graphs for n=2.

Supplementary Figure 4
**(A)** Representative flow plot of lineage negative cells from HC left untreated (Basal n=4), treated with 20% OA synovial fluid (SF n=7), or with GMCSF/IL4 (n=4) cocktail. **(B)** Histogram representing the frequency of CD209/CD14^+^ DC and frequency of the maturation markers CD40, CD80, CD83, singularly expressed. Differences among groups were evaluated by non-parametric t-test (Mann-Whitney test) *p<0.05. **(C)** Flow analysis of isolated CD209+ DC from HC left untreated (n=4) or stimulated with 20% SF from OA (n=6), displaying the frequency of the chemokine receptors CCR6, CCR7 and CXCR3. Data are represented as mean ± SEM.

Supplementary Figure 5
**(A)** Multiplex analysis of RA SF (n=16), PsA SF (n=15) and OA SF (n=11) analytes included chemokines (I-TAC, IP-10, MDC, SDF-1α, MCP-1, MCP-2, MCP-3, MCP-4, MIP-1α, MIP-1β, MIP-3α, MIP-3β) and cytokines (GM-CSF, IL-12p70, IL-1β, IL-33, IL-4, TNFα, TNFβ) and). **(B)** Differentially expressed chemokines and cytokines in RA *vs* PsA fluid. Data are represented as mean ± SEM and differences among groups were evaluated by non-parametric t-test (Mann-Whitney test) *p < 0.05.
