# Supplementary figures and images for: CD209/CD14+ Dendritic Cells Characterization in Rheumatoid and Psoriatic Arthritis Patients: Activation, Synovial Infiltration, and Therapeutic Targeting
Source: Front Immunol. 2022 Jan 12;12:722349. doi: 10.3389/fimmu.2021.722349 (PMC8789658; doi:10.3389/fimmu.2021.722349)

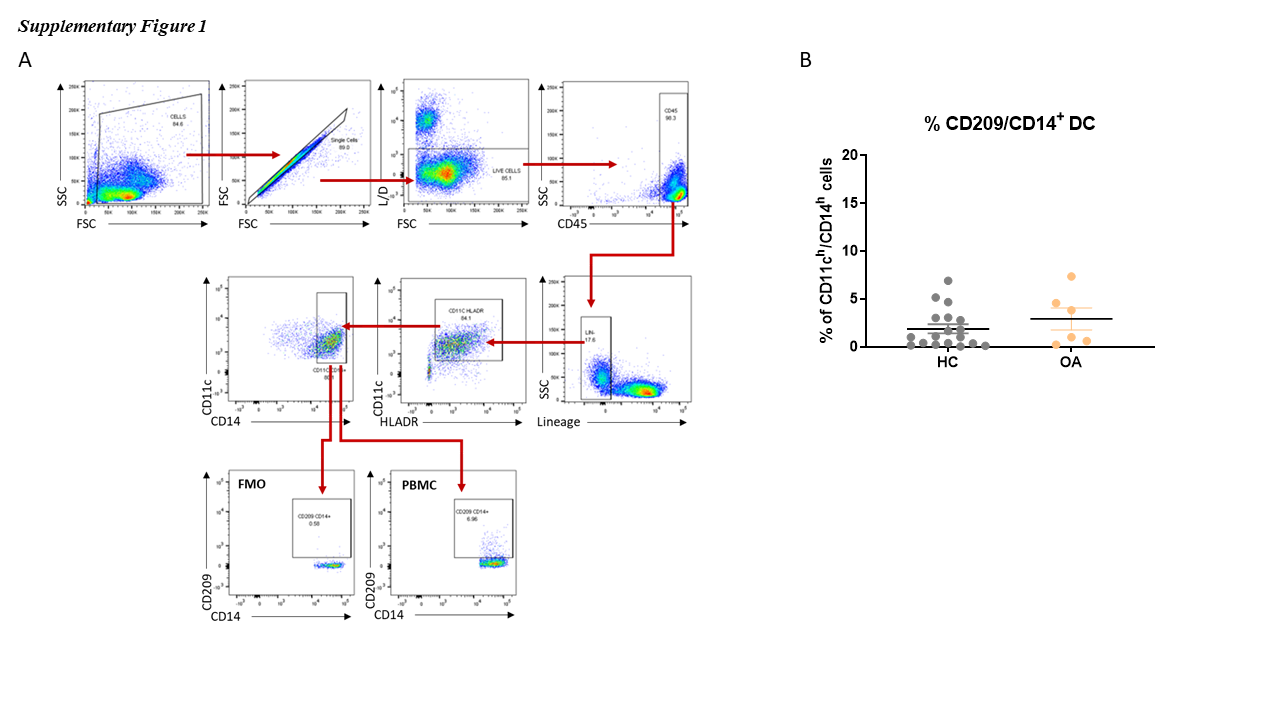

Supplement: Supplementary Figure 1 — (A) Gating strategy for CD209/CD14+ DC. Cells were gated based on forward and side scatter and dead cells and doublets were removed. Live Dead Near-IR (Molecular Probes) was used to eliminate dead cells. The cells were then gated as CD45+/LIN−(CD19-CD20-CD56-CD3)/CD11c+/HLADR+ CD14h/CD11ch CD209+/CD14+. The positive CD209/CD14+ DC population was characterized against the FMO (Fluorescence Minus One Control). (B) Representative dot-plot of frequency of CD209/CD14+ DC, showing the frequency of the parent population (CD11ch/CD14h) in HC (n=19) and OA (n=6). [file Image_1.tif]

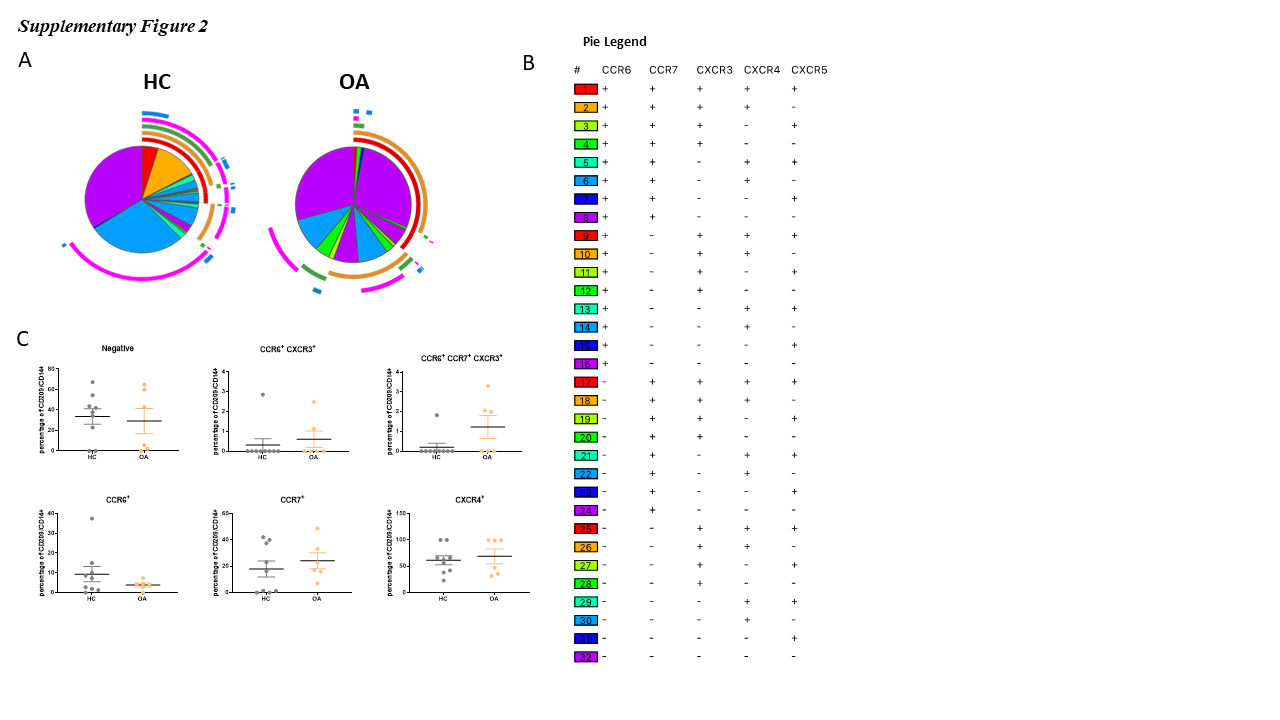

Supplement: Supplementary Figure 2 — (A) SPICE algorithm flow cytometric analysis of peripheral blood HC (n=9) and OA (n=6). CD209/CD14+ DC expression showing the frequency (represented as frequency of the parent population (CD209/CD14+)) of the chemokines receptors CCR6 (red arc), CCR7 (yellow arc), CXCR3 (green arc), CXCR4 (pink arc) and CXCR5 (blue arc) and corresponding (B) Pie Legend for all the combination of chemokine receptors. (C) Dot plot of negative population (cells not expressing chemokine receptors), CCR6+CXCR3+, CCR6+CCR7+CXCR3+, CCR6+, CCR7+, CXCR4+ expressing cells. Data are represented as mean ± SEM [file Image_2.tif]

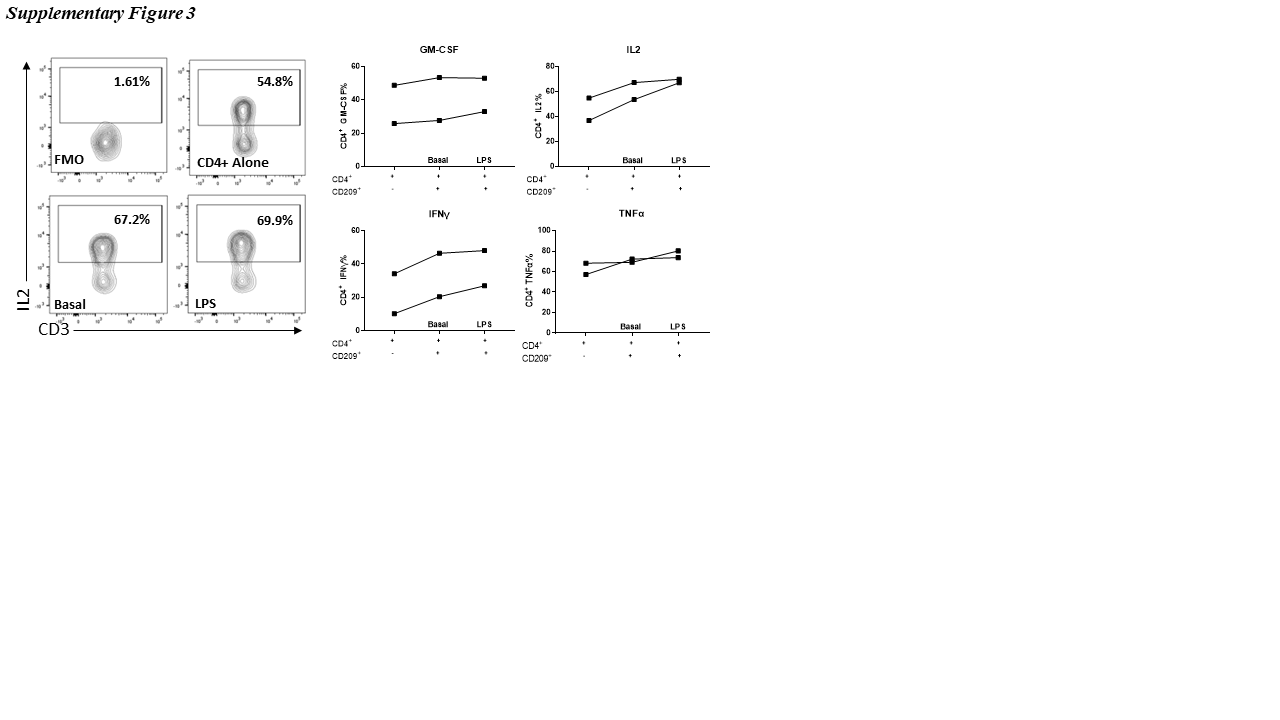

Supplement: Supplementary Figure 3 — Intracellular staining for GM-CSF, IL-2, IFN γ and TNFα expressed in synovial CD4+ T cells alone or co-cultured with synovial CD209+ DC. Left, representative flow plot. Right, Line graphs for n=2. [file Image_3.tif]

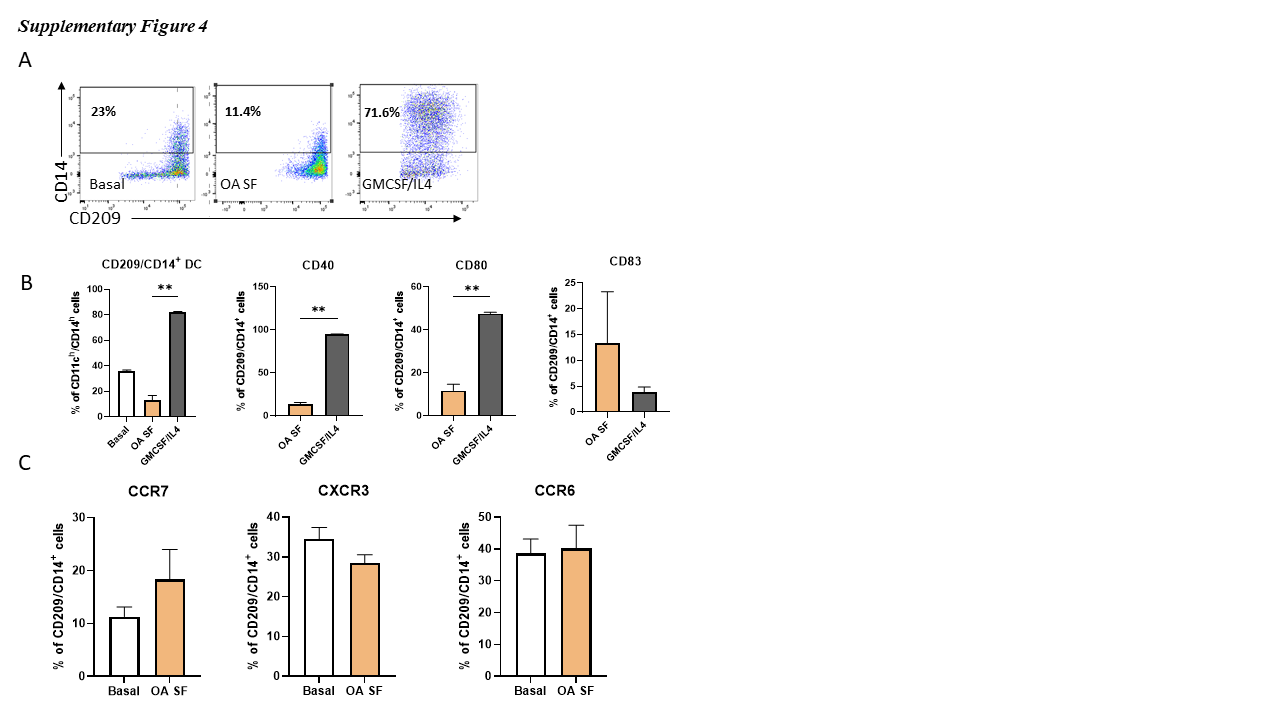

Supplement: Supplementary Figure 4 — (A) Representative flow plot of lineage negative cells from HC left untreated (Basal n=4), treated with 20% OA synovial fluid (SF n=7), or with GMCSF/IL4 (n=4) cocktail. (B) Histogram representing the frequency of CD209/CD14+ DC and frequency of the maturation markers CD40, CD80, CD83, singularly expressed. Differences among groups were evaluated by non-parametric t-test (Mann-Whitney test) *p<0.05. (C) Flow analysis of isolated CD209+ DC from HC left untreated (n=4) or stimulated with 20% SF from OA (n=6), displaying the frequency of the chemokine receptors CCR6, CCR7 and CXCR3. Data are represented as mean ± SEM. [file Image_4.tif]

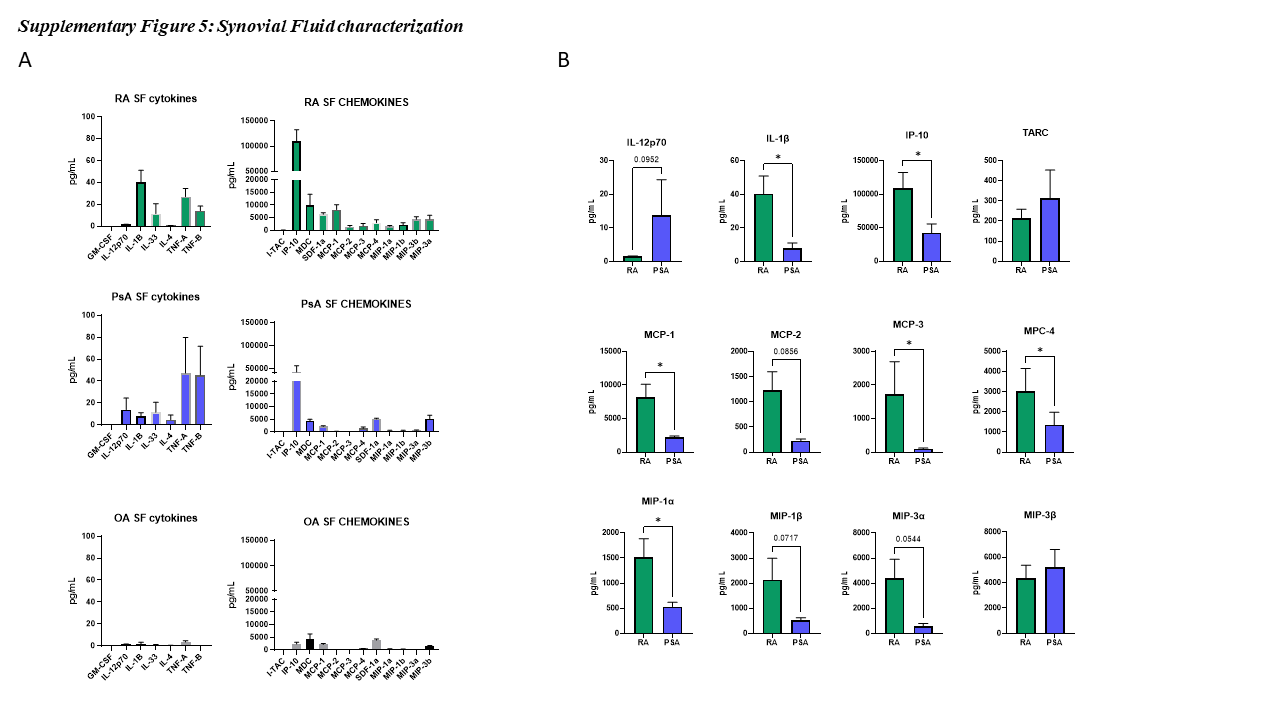

Supplement: Supplementary Figure 5 — (A) Multiplex analysis of RA SF (n=16), PsA SF (n=15) and OA SF (n=11) analytes included chemokines (I-TAC, IP-10, MDC, SDF-1α, MCP-1, MCP-2, MCP-3, MCP-4, MIP-1α, MIP-1β, MIP-3α, MIP-3β) and cytokines (GM-CSF, IL-12p70, IL-1β, IL-33, IL-4, TNFα, TNFβ) and). (B) Differentially expressed chemokines and cytokines in RA vs PsA fluid. Data are represented as mean ± SEM and differences among groups were evaluated by non-parametric t-test (Mann-Whitney test) *p < 0.05. [file Image_5.tif]
